# Structure-Activity Relationship and Mode of Action of a Frog Secreted Antibacterial Peptide B1CTcu5 Using Synthetically and Modularly Modified or Deleted (SMMD) Peptides

**DOI:** 10.1371/journal.pone.0124210

**Published:** 2015-05-21

**Authors:** Parvin Abraham, Anand Sundaram, Asha R, Reshmy V, Sanil George, K. Santhosh Kumar

**Affiliations:** Chemical Biology Laboratory, Rajiv Gandhi Centre for Biotechnology, Thiruvananthapuram, Kerala, India; Second University of Naples, ITALY

## Abstract

All life forms are equipped with rapidly acting, evolutionally conserved components of an innate immune defense system that consists of a group of unique and diverse molecules known as host defense peptides (HDPs). A Systematic and Modular Modification and Deletion (SMMD) approach was followed to analyse the structural requirement of B1CTcu5, a brevinin antibacterial peptide amide identified from the skin secretion of frog *Clinotarsus curtipes*, India, to show antibacterial activity and to explore the active core region. Seventeen SMMD-B1CTcu5 analogs were designed and synthesised by C and N-terminal amino acid substitution or deletion. Enhancement in cationicity by N-terminal Lys/Arg substitution or hydrophobicity by Trp substitution produced no drastic change in bactericidal nature against selected bacterial strains except *S*. *aureus*. But the sequential removal of N-terminal amino acids had a negative effect on bactericidal potency. Analog B1CTcu5-LIAG obtained by the removal of four N-terminal amino acids displayed bactericidal effect comparable to, or in excess of, the parent peptide with reduced hemolytic character. Its higher activity was well correlated with the improved inner membrane permeabilisation capacity. This region may act as the active core of B1CTcu5. Presence of C-terminal disulphide bond was not a necessary condition to display antibacterial activity but helped to promote hemolytic nature. Removal of the C-terminal rana box region drastically reduced antibacterial and hemolytic activity of the peptide, showing that this region is important for membrane targeting. The bactericidal potency of the D-peptide (DB1CTcu5) helped to rule out the stereospecific interaction with the bacterial membrane. Our data suggests that both the C and N-terminal regions are necessary for bactericidal activity, even though the active core region is located near the N-terminal of B1CTcu5. A judicious modification at the N-terminal region may produce a short SMMD analog with enhanced bactericidal activity and low toxicity against eukaryotic cells.

## Introduction

Short, cationic, amphiphilic peptides with antimicrobial activity are widely distributed in all life forms including humans which can kill a broad-spectrum of pathogens like bacteria, fungi, parasites, enveloped viruses and even transformed or cancerous cells with ostensibly limited chance to develop resistance [1–7]. These unique and diverse groups of molecules can be divided into various subgroups based on their amino acid composition and structure. Generally they are 12–50 residues long with significant differences in their amino acid composition and sequence. They adopt various secondary structures like α-helical, β-stranded, extended conformation and even form β-hairpin or loop structures due to the influence of disulfide bond and/or peptide chain cyclization in membrane mimicking environment [8]. Majority of them contain more number of arginine and/or lysine and histidine residues that contribute a net positive charge ranging from +2 to +9 to the peptide at neutral pH. Compared to conventional antibiotics, antimicrobial peptides (AMPs) act very fast and therefore the chances to develop resistance against them by any bacterial strain are extremely low [9]. The exact mechanism by which they kill the bacteria or the ways they exhibit toxicity against mammalian cells are not fully understood. Considering the increasing incidence of emergence of new bacterial strains with the ability to resist the action of conventional antibiotics, there is an immediate requirement for novel drug molecules [10]. AMPs are the most potent choice to design novel antibiotics to fight against bacterial infection because of their broad spectrum activity and the unique mode of action. But for therapeutic application, these molecules should be short, proteolytically stable and display high bactericidal potency, leaving human cells unaffected. A quantitative analysis of structure-activity relationship and elucidation of the mode of action using SMMD analogs may help to achieve this target.

Frog skin secretion is a complex cocktail of many pharmacologically active compounds in which AMPs are the major component [11]. These molecules are synthesized and stored in the granular glands of the dermal layer of their skin. Recently, we isolated several peptides showing antibacterial activity against a broad spectrum of bacteria from the frog *C*. *curtipes* of the Western Ghats, India which include five brevinin peptides B1CTcu1-5 [12]. A twenty-one residue peptide amide B1CTcu5 that shared significant sequence similarity with brevinin-1 family of AMPs, showed direct and potential bactericidal activity against several tested bacterial strains. Structurally, B1CTcu5 had an N-terminal hydrophobic region, a proline hinge region in the middle, and a C-terminal cyclic loop called rana box formed by the intramolecular disulphide (^15^Cys-Lys-Xaa_4_-^21^Cys) bond. Compared to the isolated other four brevinin peptides, only B1CTcu5 possess C-terminal rana box. Therefore this peptide was used as the template to study the structure-activity relationship. In the present study, we designed and synthesized seventeen SMMD analogs of B1CTcu5 by modifying various structural parameters based on the current knowledge on structural prerequisites to understand the active region responsible for its activity. This includes a D-enantiomeric peptide (D-peptide), C and N-terminal truncated and substituted SMMD-B1CTcu5 analogs. The changes induced by amino acid substitution or deletion on physico-chemical characters such as cationicity, hydrophobicity, amphipathicity and helicity that govern the overall structural features of B1CTcu5 were also examined. Though the rana box and the C-terminal disulphide bond are conserved throughout the brevinin peptides of Ranidae family, their functional and structural importance is not yet clearly understood. The C-terminal deleted and substituted analogs may help to understand the influence of disulphide-bridge and rana box on B1CTcu5 activity.

## Materials and Methods

Fmoc-amino acids, CLEAR amide resin, 2-(1H-benzotriazol-1-yl)-1,1,3,3,-tetramethyl uronium hexaflurophosphate (HBTU),4-[2,4-dimethoxyphenyl) (Fmoc-amino) methyl] phenoxyacetic acid and hydroxybenzotriazole (HOBt) were obtained from Peptide International, USA. Tri-isopropyl silane (TIS) and trifluro acetic acid (TFA), thioanisole, histopaque, ethanedithiol (EDT), NPN, SYTOX green, Polymyxin B, trifluoroethanol, (TFE) and diisopropylethylamine (DIEA) were purchased from Sigma-Aldrich Corporation, USA. [DiBAC_4_ (3)] was obtained from Molecular probes, Eugene. Piperidine and solvents (HPLC grade) were purchased from Merck, India. HPLC purification and analysis was done on a Shimadzu instrument using C18 column. Mass spectra were recorded on a MALDI-TOF mass spectrometer (AB Sciex 4800 plus). Flow cytometry analysis was carried out on a FACS Aria instrument, BD, Sparks. All other reagents used are of analytical grade procured from the local suppliers.

### Peptide synthesis

C-terminal amidated peptides were synthesized by the stepwise manual solid phase synthesis technique using CLEAR amide resin and following the 9-fluorenylmethoxy carbonyl (Fmoc) chemistry. After synthesis, the respective peptide amides were cleaved from the resin by adding a cleavage cocktail TFA/EDT/TIS/water (95:1.5:1.5:2 (v/v)) at room temperature for 4 h. The mixture was filtered and the filtrate was concentrated under vacuum to 1/4^th^ of its volume. The crude peptide amide was precipitated by ice-cold diisopropyl ether and collected by centrifugation at 3000 rpm for 15 min. After lyophilisation, the crude peptide was dissolved in solvent A (water with 0.1% TFA) and purified by injecting it into a Vydac 238 TP column (150 × 4.6 mm) and performing C18 RP-HPLC. The peptide amide was eluted out of the column by slowly increasing the acetonitrile concentration in solvent A to 70% over 30 min with 1 mL/min flow rate and monitored by setting the UV detection at 214 nm. Homogeneous purification and mass accuracy were confirmed by analytical C18 RP-HPLC and MALDI-TOF MS analysis. The cysteine oxidation was performed by stirring the peptide amide in 50% aqueous DMSO solution in the absence of sunlight. The extent of cyclisation was monitored by injecting a small volume of the peptide-folding solution at various time intervals to analytical C18 RP-HPLC and running the above mentioned gradient elution in 20 min and monitored the extent of cyclisation for 12 h..

### Circular dichroism spectroscopy

The propensity of the peptides to assume proper secondary structure was probed by CD spectroscopic analysis using a Jasco J-715 spectropolarimeter (Jasco, Tokyo, Japan). Fifty microliters of 50μg peptide was treated with 10 mM SDS micelles, taken in a quartz cell of 1 mm path length and scanned from 190–250 nm at 25°C using 1 nm bandwidth and 50 nm/min scan speed. The spectrum obtained was averaged over three consecutive scans followed by the subtraction of solvent CD signal. The mean residue ellipticity was plotted against wavelength.

### Antimicrobial activity

Bacterial strains used for *in vitro* antibacterial assay include *E*. *coli* MG1655, clinical isolates of *Vibrio cholerae* procured from the Thiruvananthapuram medical college, Kerala, India, *Staphylococcus aureus* (MTCC 9542), *Bacillus subtillis* (ATCC 14416) *Bacillus coag*ulans (ATCC 7050), *P*. *aeruginosa* (ATCC 27853), MRSA (ATCC 43300) and VRE (ATCC 29212). Bacterial cultures were grown in Luria Bertani broth (Hi-media) by overnight incubation at 37°C with constant shaking. Microbial cultures having 10^6^ CFU/ml were made from OD 0.6 culture. Respective peptide stock concentration (1mg/ml) were prepared in sterile water and diluted in LB broth to make concentrations ranging from 1–200 μg/ml. Bacterial culture (50 μl) was treated with 50 μl of individual peptide amide solution of various concentrations and incubated for 24 h at 37°C with constant shaking in a 96 well microtitre plate. Growth control wells had the same amount of bacterial inoculum but without peptide. Absorbance at 600 nm was noted in every 3 h up to 24 h to assess the cell growth and a graph was plotted with time against OD values. The minimum inhibitory concentration (MIC) was taken as the dose at which 100% of growth inhibition was observed and was calculated from the graph. MIC was calculated from independent experiments performed in triplicate.

### Antibiofilm activity

In vitro activity of the peptides was evaluated against biofilm formed by *S*. *aureus* MTCC 9542 strains. Briefly, overnight culture was diluted in LB broth (1:100) and the OD was adjusted to 0.03–0.05. Bacterial inoculum (200 μl) was taken in a 96-well flat bottom poly styrene tissue culture plate (Corning, USA) and incubated at 37°C for 12 h to grow the biofilm. Once the biofilm was formed, the wells were washed twice with 200 μl of PBS (pH 7.4) to remove any planktonic cells. Peptide (100 μg) was added to the biofilm and kept at 37°C. After 12 h, the wells were washed with PBS for three times and 100 μl of 0.1% of crystal violet was added to it and kept for 15 min at room temperature. Crystal violet was discarded, the wells were washed with PBS and dried at room temperature. DMSO (200 μL) was added to the well and the OD at 570 nm were measured using a microtiter plate reader (Biorad 680).

### Ethics statement

The blood samples required for hemolytic analysis and PBMC isolation was collected from a healthy individual with written consent after getting the formal approval from the Institutional human ethics committee of Rajiv Gandhi Centre for Biotechnology (IHEC/165/SANL/2012).

### Hemolytic assay

Hemolytic assay was carried out as previously described [13]. Briefly, 10% (v/v) suspensions of fresh human erythrocytes in phosphate buffered saline (pH 7.2) was incubated with 10 μg of peptide at 37°C for 60 min. The cells were centrifuged (3000 x g) for 5 min, and absorbance of the supernatant was measured at 595 nm. Hemolysis caused by 10% Triton X-100 was taken as positive control. Percentage hemolysis was calculated by measuring the amount of hemoglobin released as a result of erythrocytes lysis and it was calculated by performing three independent experiments.

### Preparation of peripheral blood mononuclear cells

Peripheral blood mononuclear cells (PBMCs) were isolated from heparanised blood of healthy individuals. Five milliliter of blood was diluted with PBS (pH 7.4), and layered above 5 ml of histopaque and centrifuged at 1200 rpm for 30 min at room temperature. The upper layer was discarded, and the interphase containing PBMCs were carefully withdrawn and washed with PBS. It was centrifuged at 1500 rpm at room temperature for 10 min, the pellets were resuspended in RPMI medium and the cells were counted.

### MTT assay

Cytotoxicity of the peptides against human embryonic kidney (HEK) cells, skin cancer cells (A431) and PBMCs were evaluated by 3-[4, 5-dimethylthiazol-2-yl]-2, 5-diphenyl tetrazolium bromide (MTT) assay [14]. Peptide solution prepared in less than 0.2% DMSO (10–100 μg/ml) was treated with initially seeded 10,000 cells per well for HEK and A431 cells. To assess peptide cytotoxicity in PBMCs, MTT assay was performed by adding different concentrations of the respective peptides to 96 well plate containing 40,000 PBMCs per well, in triplicate. The conversion of MTT into tetrazolium salt was determined by measuring the absorbance at 570 nm.

### Membrane permeabilisation assays

The outer membrane permeabilisation activity of the peptides was determined by the NPN assay [15]. An overnight culture of *E*. *coli* MG1655 was diluted in LB medium and grown to an A_600_ of 0.4–0.6. The cells were harvested, washed, and re-suspended in the same volume of buffer (5 mM HEPES, pH 7.2, 5 mM KCN). For NPN assay, 1mL of cells and 10 μM NPN were mixed, and fluorescence was measured using a fluorescence spectrophotometer. The increase in fluorescence with the addition of increasing concentrations of peptide was measured.

The inner membrane permeability of the bacterial cells was assayed by measuring the penetration of SYTOX green into the microbial cells. Overnight grown *E*. *coli* MG1655 culture was washed and resuspended in PBS. Cells of OD 0.4–0.6 were taken in PBS and incubated at 37°C with 1 μM SYTOX green in the absence of light for 15 min. The increase in fluorescence was measured as a function of peptide concentration at an excitation wavelength of 485 nm and emission wavelength of 520 nm.

### Flow cytometry

*E*. *coli* cells were incubated with peptides at their respective MICs for 1 h at 37°C and treated with the dye bis-(1,3-dibutylbarbituric acid) trimethine oxonol [DiBAC_4_ (3)] (1 μg/ml). After 10 min incubation, cell suspension was centrifuged (5000 x g) for 8 min and the pellet obtained was suspended in 1 ml PBS. Peptide induced bacterial membrane depolarization was measured flow cytometrically using argon laser at excitation wavelength 490 nm and the emission maximum at 516 nm [16]. The green fluorescence in the channel FL1 was measured. A total of 10,000–50,000 events were analysed in each sample. The data acquisition and analysis were performed with the help of DIVA software (BD). The Forward Scatter Side Scatter-Dot Plot referring to relative cell size and granularity of bacterial population were differentiated from the background signals and gated for evaluation of the fluorescence. A marker was drawn to gate the viable cells in the control.

## Results

### Design and synthesis of B1CTcu5 analogs

SMMD-B1CTcu5 analogs that are designed and synthesized are given in Table 1. Peptides were purified by RP-HPLC using C18 column and their purity and identity were confirmed by MALDI-TOF MS analysis. Since increase in cationicity is a robust strategy to improve the antibacterial property of AMPs, B1CTcu5K and B1CTcu5R was synthesized by substituting the N-terminal Leu with cationic Lys/Arg. According to hydrophobicity scale [17], the mean hydrophobicity value of Trp (32.4) is greater than that of Leu (23.3). Therefore the effect of increase in overall hydrophobicity of B1CTcu5 on bactericidal activity was analysed by substituting the N-terminus Leu with Trp to get B1CTcu5W. B1CTcu5H was synthesised by substituting the N-terminal Leu with His to understand the effect of a polar residue at the N-terminus. Importance of the N-terminal amino acid residues of B1CTcu5 on activity was further analysed by their sequential removal from the N-terminal. B1CTcu5-L was synthesized by removing the N-terminal Leu. Similarly other deletion peptides B1CTcu5-LI, B1CTcu5-LIA, and B1CTcu5-LIAG were synthesized by the sequential removal of N-terminal LI, LIA and LIAG. B1CTcu5LGG is a twenty-one residue SMMD analog designed to analyse the importance of the effective length of the peptide and the positional importance of N-terminal Leu on activity. Here the second and third residues Ile and Ala were substituted with two Gly residues to keep the overall length of the peptide similar to that of the parent peptide. The influence of C-terminal intramolecular disulphide bridge on activity was studied by blocking its formation either by knocking off the C-terminal Cys residue to get B1CTcu5-C or by substituting the 15^th^ residue Cys with TFA stable side chain protected Cys(Acm) to get B1CTcu5C(Acm). The role of C-terminal end and the cyclic loop rana box was analysed by removing six C-terminal residues to get B1CTcu5-Rana. The ten residue N-terminal half B1CTcu5L_1_-L_10_ was synthesized by removing eleven C-terminal amino acids and B1CTcu5P_11_-C_21_ was synthesized by removing ten N-terminal amino acids. DB1CTcu5 was synthesized using D-amino acids to study whether the peptide-bacterial membrane interaction is stereospecific in nature.

**Table 1:** Primary sequences of SMMD B1CTcu5 analogs.

Peptide name	Sequences	No of residues	Charge	% hydrophobicity
linear B1CTcu5	L IAGLA ANFLP QILCK IARKC	21	3	65
Cyclic B1CTcu5	L IAGLA ANFLP QILCK IARKC	21	3	66
B1CTcu5-L	IAGLA ANFLP QILCK IARKC	20	3	65
B1CTcu5-LI	AGLA ANFLP QILCK IARKC	19	3	63
B1CTcu5-LIA	GLA ANFLP QILCK IARKC	18	3	61
B1CTcu5-LIAG	LA ANFLP QILCK IARKC	17	3	64
B1CTcu5K	K IAGLA ANFLP QILCK IARKC	21	4	61
B1CTcu5W	WIAGLA ANFLP QILCK IARKC	21	3	66
B1CTcu5R	R IAGLA ANFLP QILCK IARKC	21	4	61
B1CTcu5H	HIAGLA ANFLP QILCK IARKC	21	3	61
B1CTcu5LGG	LGGGLA ANFLP QILCK IARKC	21	3	57
B1CTcu5-C	L IAGLA ANFLP QILCK IARK	20	3	65
B1CTcu5C (Acm)	L IAGLA ANFLP QILC(Acm)KIARKC	21	3	66
B1CTcu5-Rana	L IAGLA ANFLP QILC	15	0	73
B1CTcu5L1-L10	LIAGL A ANFL	10	0	80
B1CTcu5P11-C21	P QILCK IARKC	11	3	54
DB1CTcu5	L IAGLA ANFLP QILCK IARKC	21	3	66

### Secondary structure analysis

**Fig 1:**
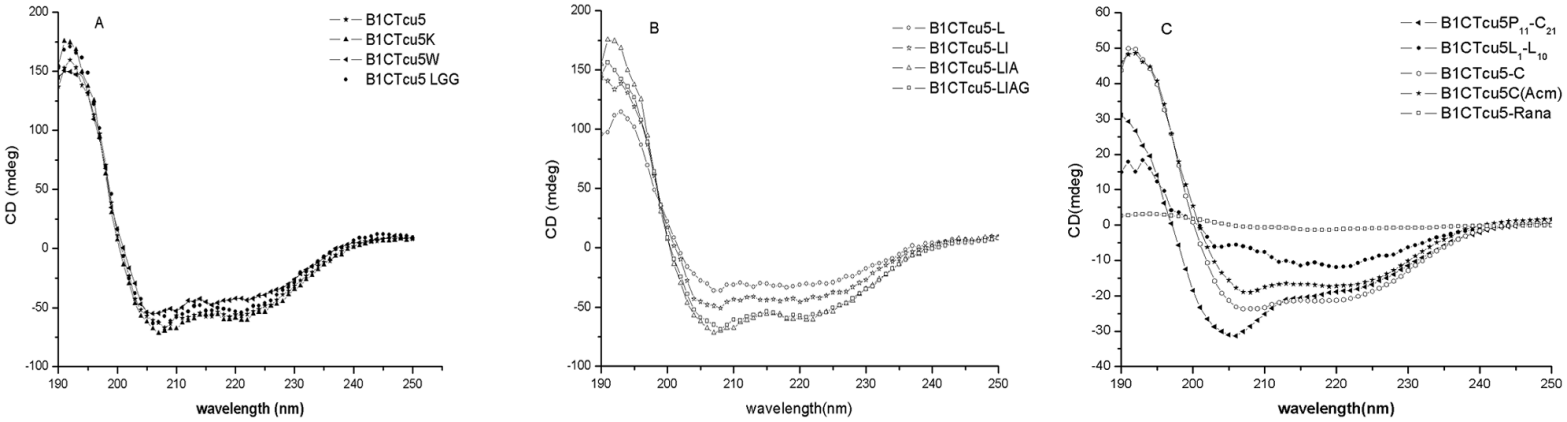
CD spectra of B1CTcu5 and analogs. 50 μg of peptides were analysed by treating with 10 mM SDS micelles. The spectra were averaged over three consecutive scans and the solvent CD signal was subtracted.

B1CTcu5 and its analogs except B1CTcu5-Rana and B1CTcu5L_1_-L_10_ produced a negative band in the CD spectra centered at 208 and 222 nm and a positive band centered at 193 nm, indicating these SMMD peptides adopted significant α-helical conformation in SDS micelles (Fig 1). The linear B1CTcu5 also formed a proper secondary structure but B1CTcu5-C showed only a very low propensity to form a proper alpha helical conformation. Spectral analysis also showed that N-terminal substitution or deletion do not influence the propensity to form a stable α-helical conformation in SDS micelles. But in aqueous solution these peptide showed no preferential secondary conformation.

### Antimicrobial activity and hemolysis

B1CTcu5 and its analogs were tested for antibacterial property against several gram-positive and negative bacterial strains and the results are summarized in Table 2. A single N-terminal Lys substitution was not good enough to produce a drastic change in bactericidal potency compared to the parent peptide except against *S*. *aureus*, where the MIC was improved from 100 to 12.5 μg/ml. This substitution helped to reduce the hemolytic property of B1CTcu5 from 40 to 15% (Table 2). N-terminal Arg substitution helped to reduce the MIC against *S*. *aureus* and MRSA from 100 to 25 μg/ml and against *V*. *cholerae* from 25 to 12.5 μg/ml. The substitution also brought down hemolytic potential from 40 to 17.4%. Enhancement of peptide hydrophobicity by N-terminal Trp substitution helped to augment the bactericidal potency of the peptide against *S*. *aureus*, without affecting the hemolytic potential. N-terminal His substitution did not produce any change in antibacterial character of the analog compared to B1CTcu5 but observed a 10% reduction in hemolytic nature. The analog produced by the removal of N-terminal Leu, though abridged the MIC against *S*. *aureus* by about four-fold, its effectiveness against other tested bacterial strains were less compared to the parent peptide. The decrease in antibacterial activity directly depended upon the number of residues eliminated from this end. The analog B1CTcu5-LIAG obtained after removing four N-terminal amino acids was the most potent peptide among all synthetic SMMD analogs, especially against *S*. *aureus* where it showed MIC as low as 6.25 μg/ml. This deletion also reduced the hemolytic potential by about 10% compared to B1CTcu5. Elimination of six C-terminal residues (rana box) completely abolished the antibacterial and hemolytic nature of B1CTcu5.

**Table 2:** Antibacterial and hemolytic activity of synthetic B1CTcu5 and SMMD analogs.

				Microorganisms					
Peptides	*B*. *coagulans* ATCC 7050	*B*. *subtilis* MTCC 14416	*S*. *aureus* ATCC 9542	*E*. *coli* MG 1655	*P*. *aeruginosa* ATCC 27853	*V*. *cholerae*	MRSA ATCC 43300	VRE ATCC 29212	Hemolysis (%)
				MIC(μg/ml)^a^					
Linear B1CTcu5	12.5	50	>100	25	>100	25	>100	100	37
Cyclic B1CTcu5	15	50	>100	25	>100	25	>100	100	40
B1CTcu5-L	100	100	25	50	>100	25	>100	NA	30.5
B1CTcu5-LI	80	NA	50	50	>100	25	NA	NA	26
B1CTcu5-LIA	100	100	50	100	>100	50	NA	NA	26.5
B1CTcu5-LIAG	25	50	6.25	12.5	>100	12.5	50	100	30
B1CTcu5K	12.5	100	12.5	25	>100	25	>100	50	15
B1CTcu5W	12.5	50	12.5	100	>100	25	25	50	39.11
B1CTcu5R	12.5	50	25	25	>100	12.5	25	50	17.4
B1CTcu5H	25	50	>100	50	>100	25	100	NA	30.4
B1CTcu5LGG	NA	NA	NA	NA	NA	NA	NA	NA	23.4
B1CTcu5-C	25	50	50	25	>100	50	50	50	13
B1CTcu5C (Acm)	25	50	50	12.5	>100	50	50	100	25
B1CTcu5-Rana	NA	NA	NA	NA	NA	NA	NA	NA	NA
B1CTcu5L1-L10	NA	NA	NA	NA	NA	NA	NA	NA	NA
B1CTcu5P11-C21	NA	NA	NA	NA	NA	NA	NA	NA	NA
DB1CTcu5	15	50	50	25	>100	NA	25	50	40.22

^a^—MIC represents the lowest peptide concentration required to kill entire bacteria, NA-not active up to the highest concentration tested.

The influence of C-terminal intramolecular disulphide bridge was more obvious in hemolytic activity than on antimicrobial potency. Blocking the disulphide bond formation either by the removal of C-terminal Cys residue or by the substitution of Cys(Acm) resulted two linear peptides. Both the analogs showed more or less same activity compared to the parent peptide indicating the insignificance of disulphide-bridge on antibacterial activity of B1CTcu5. Removal of the disulphide bridge also helped to lower the hemolytic nature of the peptide. Analysis of the influence of N-terminal Leu residue on peptide chain length, and its position on activity with the help of SMMD-analog B1CTcu5LGG was not informative as the analog was inactive. The substitution of N-2 Ile and N-3 Ala with Gly totally abolished the antibacterial nature of the peptide. Both the N-terminal half B1CTcu5L_1_-L_10_ and the C-terminal half B1CTcu5P_11_-C_21_ are inactive against all tested bacterial strains and RBCs. The bactericidal potency of D-peptide DB1CTcu5 was almost the same as the parent peptide.

### Action of peptides on biofilm

**Fig 2:**
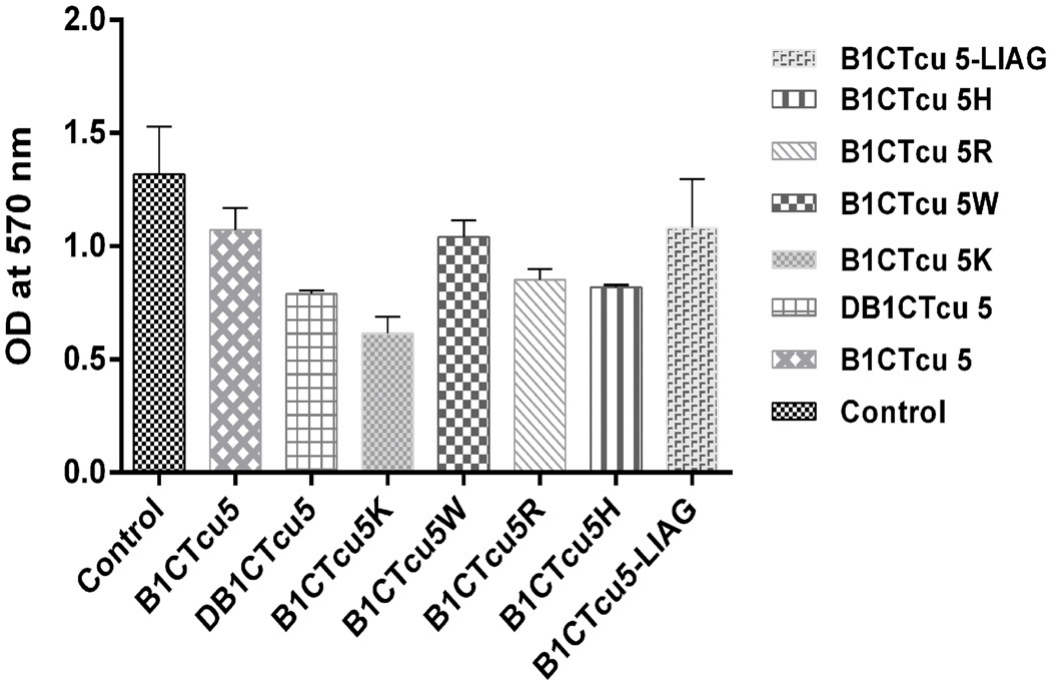
Effect of peptides against biofilm formation by *S*. *aureus* strains. Biofilm formed by *S*. *aureus* at 12 h were treated with 100μg/ml of peptides for 12 h and the biofilm formation was determined by measuring the absorbance at 570 nm.

The antibiofilm activity of the potent peptides was as evaluated on *S*. *aureus* strains (same strain used for antibacterial analysis) and the results are presented in Fig 2. B1CTcu5K exhibited 54.3% biofilm inhibition activity while the parent peptide B1CTcu5 showed only 18.6% compared to untreated bacterial cells. The percentage inhibition showed by the SMMD analogs DB1CTcu5, B1CTcu5W, B1CTcu5R, B1CTcu5H and B1CTcu5-LIAG are 34.25%, 13%, 31.21%, 32.5% and 25% respectively.

### Cell viability assay

The concept of selective toxicity of the SMMD peptides against tumorous A431 cell lines, normal HEK cell lines and human peripheral blood mononuclear cells (PBMC) were assessed by MTT assay.. No detectable level of toxicity was observed in A431 as well as HEK cells after treatment with B1CTcu5 and its analogs up to 100 μg/ml (Table 3). The peptides B1CTcu5, DB1CTcu5, B1CTcu5W, B1CTcu5R and B1CTcu5H showed cytotoxicity (CC50) against PBMCs at high concentrations while B1CTcu5K and B1CTcu5-LIAG were not cyotoxic even at 100 μg/ml (Table 3).

**Table 3:** Cytotoxicity in human cells.

	CC 50(μg/mL)		
Peptide	PBMCs	A431	HEK
B1CTcu5	75.1 ± 1.46	>100	>100
B1CTcu5D	71.4 ± 2.3	>100	>100
B1CTcu5R	96.1 ± 1.22	>100	>100
B1CTcu5H	90 ± 2.56	>100	>100
B1CTcu5K	>100	>100	>100
B1CTcu5W	74.5 ± 2.44	>100	>100
B1CTcu5-LIAG	>100	>100	>100

The values represent averages ± s.d from three separated experiments.

### Membrane permeabilisation assays

**Fig 3:**
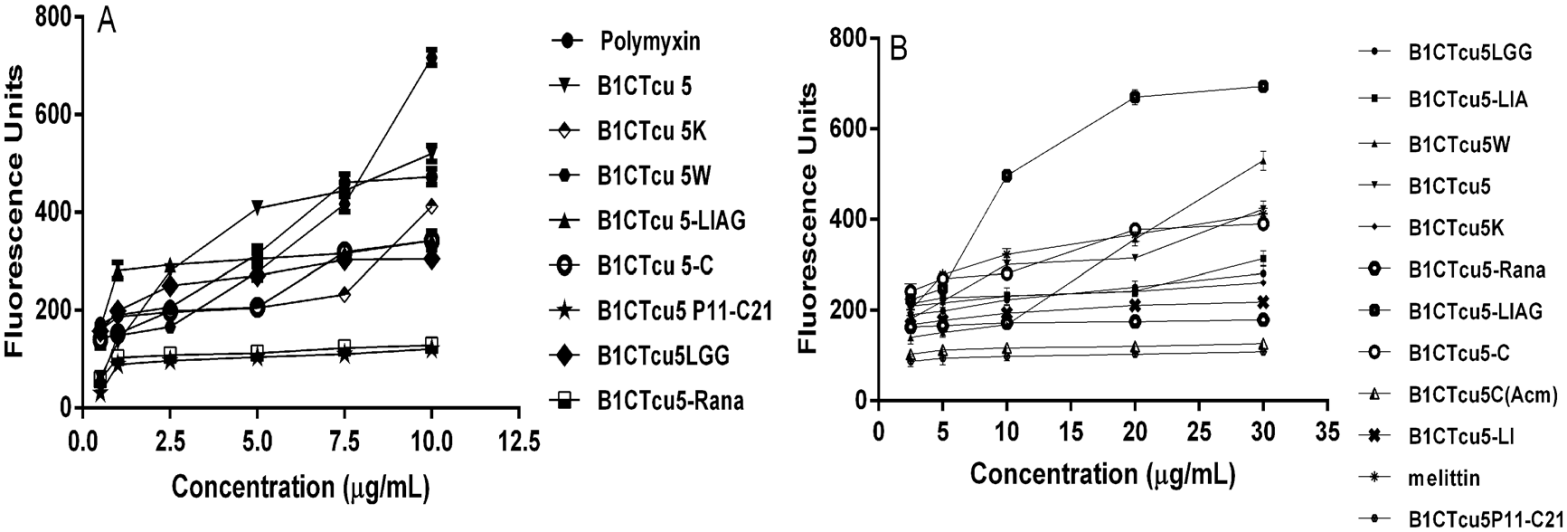
Outer and inner membrane permeabilisation in *E*. *coli* cells. (A) *E*. *coli* cells(OD 0.5–0.6) were washed with 5mM HEPES, pH 7.2 and 5mM KCN. 1mL cells were treated with 10mM NPN and increasing concentrations of peptides were added. The uptake of NPN was noted by increase in fluorescence at an excitation of 350nm and emission of 420nmas a measure of outer membrane permeabilisation. (B) Inner membrane permeabilisation efficiency of the peptides were assayed by treating *E*. *coli* cells (OD 0.4–0.6) in PBS with 1μM SYTOX green at 37°C for 15 min. Fluorescence was measured at an excitation wavelength of 485nm and emission wavelength of 520nm after peptide addition.

1-N-phenylnapthylamine (NPN) is a neutral hydrophobic probe that is normally excluded by an intact outer membrane, but can penetrate into the cell upon membrane disruption by membrane active peptide like polymyxin B and produces an intense fluorescence. The NPN uptake was observed when treated with SMMD-B1CTcu5 analogs except in the case of B1CTcu5-Rana and B1CTcu5P_11_-C_21_. The effects induced by these peptides was observed at concentrations well below their respective MICs (Fig 3A).

The peptide uptake through cytoplasmic membrane was evaluated by measuring the binding affinity of SYTOX green with intracellular nucleic acids (Fig 3B). SYTOX green is a cationic nucleic acid binding dye that cannot enter the cytoplasm, unless the cytoplasmic membrane is disturbed. Therefore the extent of cytoplasmic membrane damage caused by a peptide can be studied by measuring the fluorescence intensity produced by SYTOX green. B1CTcu5-LIAG though showed only a moderate ability to permeabilise the outer membrane; its presence resulted a dramatic increase in fluorescence intensity when treated with SYTOX green. The inner membrane permeabilisation induced by B1CTcu5-LIA was about three-fold less compared to that induced by B1CTcu5-LIAG.

### Membrane depolarization assay

**Fig 4:**
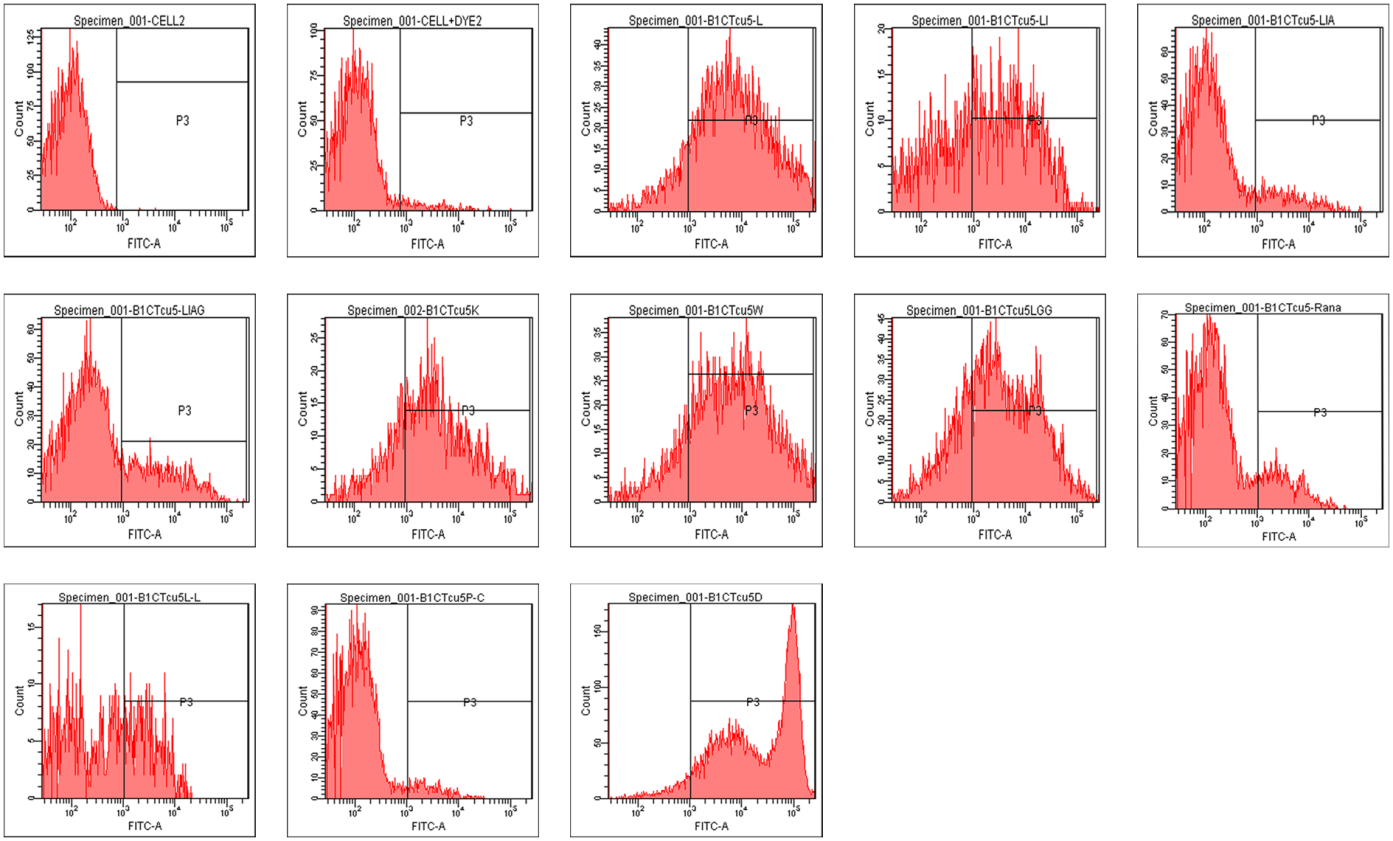
Membrane depolarization of *E*. *coli* by B1CTcu5 and its analogs. *E*. *coli* cells (10^6^ CFU/ml) were treated with peptides at their MICs for 1h and treated with 1μg/ml of [DiBAC_4_ (3)].10,000 events were selected for the assay and the bacteria were differentiated from background signals. Membrane depolarization is indicated by a shift in the population.

The ability of the peptide to enforce depolarization in bacterial cell membrane was analysed flow cytometrically and the results are given in Fig 4. Analysis showed that B1CTcu5, DB1CTcu5, B1CTcu5W, B1CTcu5K and B1CTcu5LGG are capable of inducing membrane depolarization while B1CTcu-LI produced only a partial membrane depolarization. The analogs resulted by the removal of N-terminal amino acids produced a steady decrease in their depolarization ability and it depended upon the number of residues removed. Similarly the C-terminal deletion or the removal of rana box region drastically diminished the depolarization ability. The membrane depolarization induced by the D-peptide DB1CTcu5 was almost similar to that of L-peptide.

## Discussion

B1CTcu5 is a 21-residue peptide amide isolated from the skin secretion of *C*. *curtipes*. This hemolytic peptide showed bactericidal activity against Gram-negative bacteria like *E*. *coli*, clinical isolates of *V*. *cholerae* and Gram-positive bacteria like *B*. *coagulans*, *B*. *subtilis*, VRE and MRSA. Seventeen SMMD-B1CTcu5 analogs were designed and synthesized to study the importance of primary and secondary structure on activity and to identify the pharmacophore region of B1CTcu5. These peptides were designed by swapping predictable factors that govern the antimicrobial and cytotoxic character of a peptide, which include cationicity, hydrophobicity, amphipathicity and helicity.

For exerting antibacterial property, a peptide should possess optimum balance between cationicity and hydrophobicity. This balance is important for the effective initial interaction of the peptide with the bacterial membrane. Increase in cationicity can promote their interaction with anionic phospholipids and the negatively charged lipopolysaccharides of the bacterial cell membrane [18]. Previous MD simulation studies with magainin-2 has showed that Lys substitution promoted the initial binding of a peptide with bacterial membrane interfaces by forming H-bonds, either with the phosphate oxygen atoms or with the glycerol oxygen atoms of the lipid head groups. The methylene side chain in Lys can help to enhance the interaction efficiency by penetrating deep into the membrane core region [19]. In B1CTcu5, augmentation of the cationicity by N-terminal Lys substitution produced an analog, which showed a better activity against *S*. *aureus*. This substitution also helped to reduce the hemolytic potential of the peptide. The overall reduction in hydrophobicity by the Lys substitution may have contributed to the lowering of hemolytic potential. N-terminal substitution with Arg can also enhance the overall cationicity of the peptide and make it capable of forming H-bonded interaction with the anionic component of the bacterial membrane interface. This substitution was particularly effective against *S*. *aureus* and helped to reduce the hemolytic potential of the peptide.

The overall hydrophobicity of a peptide is the measure of its ability to move from an aqueous environment to a hydrophobic phase and it influenced the peptide affinity towards the lipid acyl chains both in the model and biological membranes [20]. Trp residue can interact strongly with the membrane interfacial region by anchoring the peptide to the bilayer surface [21]. An increase in hydrophobicity by a single Trp substitution at the N-terminal of B1CTcu5 though helped to improve the MIC against *S*. *aureus*, this single mutation was not enough to produce drastic effect on its antibactericidal and the hemolytic nature of the peptide. Enhancement in polarity by N-terminal His substitution is not enough to influence the overall antibacterial and hemolytic potential of B1CTcu5H. Similarly, antibacterial character of an AMP cannot be maintained by simply keeping the peptide length or integrity of the N-terminal residue and its conformation. Substitution of amino acids at the penultimate and anti-penultimate positions can also seriously affect the antibacterial and hemolytic nature of a peptide as showed by SMMD analog B1CTcu5LGG. The sequential removal of amino acids from the N-terminal of an AMP can steadily deplete the antibacterial and hemolytic character and the decline in activity was increased with the number of residues eliminated. But the 17-residue B1CTcu5-LIAG analog resulted by the removal of four N-terminal residues showed antibacterial activity similar to, or in excess to that of parent peptide. This could be the active core region of B1CTcu5 responsible for its antibacterial property.

The structural and functional importance of the C-terminal cyclic looped rana box in brevinin-1 peptides of the *Ranidae* family is not yet clearly understood [22]. Amino acids that contributed to the net positive charge (two Lys and one Arg) that are responsible for maintaining the stability of B1CTcu5 are located in this cyclic region. Amino acid deletions from this region thus increase the net hydrophobicity of the peptide and produce inactive analogs. Previous studies has shown that the presence of disulphide bond in bactenecin is crucial for its activity and played a distinct role during its initial interaction with the outer membrane [15]. But the structure-activity analysis of AMPs like esculentin-1, gaegurin-4 and ranalexin showed that rana box region is not that important for activity [23–26]. A comparative analysis of antibacterial activity between the linear B1CTcu5, B1CTcu5-C, B1CTcu5Cys(Acm) with the cyclic B1CTcu5 showed that the presence of disulphide bond in brevinin peptides is not a necessary condition for their activity. The RP-HPLC elution profile showed that C-terminal cyclisation enhanced the overall hydrophobicity of the peptide, which in turn contributed to its enhanced hemolytic nature. Our data revealed that N-terminal hydrophobic residues and C-terminal cationic residues are equally responsible for maintaining the antibacterial nature of brevinin-1 peptides.

The ability of biofilm formation of bacteria is considered as a serious threat contributing to the pathogenesis and antibiotic resistance [27]. Since AMPs are considered as an important template for the development of alternative therapeutics, the ability of B1CTcu5 analogs to control bio-film formation was tested. B1CTcu5K reasonably destroyed the bio-film formed by *S*. *aureus*. Since the bacteria that persist within biofilm usually exhibit distinct phenotype from planktonic cells, it is not surprising that the SMMD analogs that showed potent activity against planktonic cells failed to show antibiofilm or biofilm associated bacterial killing activity.

CD spectroscopic analysis of B1CTcu5 and its analogs also showed that C-terminal residues played a significant role to stabilize the formation of alpha helical conformation. In brevinin-1E the intra-disulphide bridge is reported to induce the formation of an alpha helix [28]. Any modification in this region, as in the case of B1CTcu5-C or B1CTcu5-Rana can affect the formation of helical conformation. Ability of the C-terminal eleven-residue B1CTcu5P_11_-C_21_ to form helical conformation and the poor ability of the N-terminal ten-residue B1CTcu5L_1_-L_10_ to adopt the helical conformation further support this finding. The SMMD analog B1CTcu5LGG, though inactive against tested bacterial strains, attained a proper alpha helical conformation in micelles. The formation of a proper secondary structure in bacterial membrane is regarded as an important step for the effective peptide-membrane interaction that leads to the membrane disintegration. But this study could not draw a definite correlation between the extent of helical conformation on antibacterial activity. Studies conducted in brevinin-1E and fallaxin peptides [29] also confirmed that ranking of antimicrobial nature of peptides in terms of their secondary structure alone is not possible. The concept of B1CTcu5 containing D-amino acids was applied to study the chemical basis of peptide-membrane interaction. The bactericidal nature of B1CTcu5D showed that the D-amino acid replacement has no effect on its ability to bind to the bacterial membrane and induce the disintegration of bacterial cell membrane.

To exert its action, AMP should penetrate through the outer membrane barrier and reach the cytoplasmic membrane. Outer membrane of the bacteria usually acts as a selectively permeable barrier and it limits the accessibility of hydrophobic compounds. B1CTcu5 and its analogs showed the outer membrane permeability at concentrations well below their respective MICs. The higher activity exhibited by B1CTcu5-LIAG and the very low activity exerted by B1CTcu5-Rana can be well correlated with their membrane permeabilisation capacities, especially with the inner bacterial membrane. However, the reason for inactive antibacterial nature of B1CTcu5LGG is not clear even though it showed very good membrane permeabilisation ability.

In bacteria, the formation of trans-cytoplasmic membrane proton motive force is a major component of the energy generating mechanisms. Since the antimicrobial peptides are membrane acting, we tried to correlate membrane depolarization induced by a peptide with bacterial cell death. Though the N-terminal truncation steadily decreased the membrane depolarisation capacity of the peptides, it was not in the order of their activity. The poor depolarization showed by B1CTcu5-LIAG and good depolarization exhibited by the inactive analog B1CTcu5LGG further supports this finding.

Our studies using SMMD analogs showed that the bactericidal nature of B1CTcu5 is mainly attributed to its N-terminal region. The antibacterial pharmacophore region resides within the N-terminal region of the peptide. The C-terminal amphiphilic region may be responsible for providing the required conformational stability to the peptide and reduced sensitivity to proteolytic cleavage. Therefore both the C and N-terminal regions are important for the contribution of peptide activity and a proper balancing of cationicity, hydrophobicity, amphipathicity and helicity is important to show optimum antibacterial character.
